# Universal health care and equity: evidence of maternal health based on an analysis of demographic and household survey data

**DOI:** 10.1186/s12939-015-0184-9

**Published:** 2015-06-16

**Authors:** Sarah Neal, Andrew Amos Channon, Sarah Carter, Jane Falkingham

**Affiliations:** Department of Social Statistics and Demography, University of Southampton, Building 58, Highfield Campus, Southampton, SO17 1BJ UK; ESRC Centre for Population Change, University of Southampton, Highfield Campus, Southampton, SO17 1BJ UK

**Keywords:** Maternal health, Universal health coverage, Sustainable development goals, Monitoring, Inequity

## Abstract

**Introduction:**

The drive toward universal health coverage (UHC) is central to the post 2015 agenda, and is incorporated as a target in the new Sustainable Development Goals. However, it is recognised that an equity dimension needs to be included when progress to this goal is monitored. WHO have developed a monitoring framework which proposes a target of 80 % coverage for all populations regardless of income and place of residence by 2030, and this paper examines the feasibility of this target in relation to antenatal care and skilled care at delivery.

**Methodology:**

We analyse the coverage gap between the poorest and richest groups within the population for antenatal care and presence of a skilled attendant at birth for countries grouped by overall coverage of each maternal health service. Average annual rates of improvement needed for each grouping (disaggregated by wealth quintile and urban/rural residence) to reach the goal are also calculated, alongside rates of progress over the past decades for comparative purposes.

**Findings:**

Marked inequities are seen in all groups except in countries where overall coverage is high. As the monitoring framework has an absolute target countries with currently very low coverage are required to make rapid and sustained progress, in particular for the poorest and those living in rural areas. The rate of past progress will need to be accelerated markedly in most countries if the target is to be achieved, although several countries have demonstrated the rate of progress required is feasible both for the population as a whole and for the poorest.

**Conclusions:**

For countries with currently low coverage the target of 80 % essential coverage for all populations will be challenging. Lessons should be drawn from countries who have achieved rapid and equitable progress in the past.

## Introduction

As national and international policy makers seek to address the unfinished Millennium Development Goal (MDG) agenda as well as develop new goals and indicators to guide development there has been growing demand to place universal health coverage (UHC) as a central pillar for such efforts [1]. As a result, one of the targets linked to Goal 3 of the newly developed Sustainable Development Goals (Ensure healthy lives and promote well-being for all at all ages) is to “achieve UHC, including financial risk protection, access to quality essential health care services, and access to safe, effective, quality, and affordable essential medicines and vaccines for all.” UHC has been defined as all people receiving quality health services that meet their needs without being exposed to financial hardship [2]. Achieving this goal requires progress in three dimensions: expanding essential health services, increasing access to a greater proportion of the population and reducing out-of-pocket payments [3].

The MDGs have been justly criticised for failing to take into account issues of equity when monitoring progress (e.g.,[4]). There is strong commitment that equity is “hard-wired” into any post-MDG Goals and strategies, which was embodied by the High Level Panel of Eminent Persons in their acknowledgement that future development agenda’s must “leave no person behind” [5]. A recent framework for monitoring global progress towards UHC produced by the World Health Organisation (WHO) and World Bank stated that the overall target was to be “By 2030, all populations, independent of household income, expenditure or wealth, place of residence or gender, have at least 80 % essential health services coverage.” In order to effectively monitor this, the framework also declares that “all measures should be disaggregated by socioeconomic and demographic strata in order to allow assessment of the equitable distribution of service and financial protection coverage” [2].

Women and children are among those groups with most to gain from UHC as they are greatly affected by inequalities in access to health care. The package of care offered by UHC in most countries usually includes a set of basic preventive and curative child and reproductive health services, including access to care for pregnant women before, during and after birth [6]. Maternal health care is a key aspect of service provision in its own right, but it can also be seen as a marker for wider health systems function. Ensuring universal access to maternal health care requires the provision of a continuum of care before, during and after birth provided by a suitably trained health care provider. The majority of middle and lower income countries have achieved an increase in the percentage of women receiving antenatal care, skilled care at birth and postnatal care since 1990, but for many countries universal coverage is still a distant goal. Countries that have achieved high coverage of maternal health care from a relatively low baseline over the last three decades have progressed through a common pathway, whereby coverage has increased first among the urban rich, followed by the rural rich and the urban poor, with access among the rural poor the last to be achieved [7]. The way that inequalities in access to care have developed between socio-economic groups clearly demonstrates the inverse care law, where those who need the most care are the least likely to receive it [8, 9]. However, these inequities and the processes by which they change and develop over time are masked by indicators that measure overall population coverage for an intervention.

For many countries reaching 80 % coverage for these maternal health interventions even at the national aggregate level will be extremely challenging: in some progress has been poor to date, and many countries will be starting their journey towards UHC from a low baseline. Adding an additional dimension of equity produces further challenges: a Lancet review of 12 maternal and child health interventions found that skilled attendance was the least equitable [10], and others studies suggest inequalities for this indicator may be particularly persistent [11]. Differences between urban and rural residents are also particularly marked for skilled attendance [12].

This study examines the feasibility of the target of 80 % coverage for all populations regardless of income and place of residence outlined in the WHO framework for monitoring progress to universal coverage [3] with regards to antenatal care and skilled care at delivery. It initially presents empirical analysis of the gap that exists between current rates of coverage of antenatal care and skilled care at birth and the 80 % target by wealth quintile and urban/rural residence for 35 countries based on analysis of Demographic and Household Survey (DHS) data. In order to highlight how inequalities and required progress differ by current levels of average service uptake, we have grouped countries into very low, low, medium, high and very high coverage based on current levels of care. We then analyse the progress that will need to be made if these groups are to reach the target of 80 % coverage by 2030, and how this would differ by quintile and place of residence. We compare required progress with past progress to ascertain if the rate of progress needed to meet the target is unprecedented and, potentially, unrealistic. We then present findings of socioeconomic differentials disaggregated by urban and rural residence to demonstrate how these dimensions interact, and illustrate the need for these elements to be considered concurrently in order to have a more accurate and nuanced understanding of their impact on coverage. Our discussion will then examine current discourse about whether inequality is inevitable on the pathway to UCH, and potential measures that would make good markers of equity within a country.

### Data and methods

Data from DHS surveys were used as they are large, nationally representative surveys providing information on a range of health care indicators that are normally comparable across time and place [13]. We included all countries where three surveys were available, with the first and last at least 10 years apart, and with the earliest dating from after 1990 and the latest after 2005. A total of 35 countries met these criteria (see Appendix: Table 6 for a list of countries and survey years). Data on economic status are provided by asset wealth indices, which are constructed using principle component analysis using information on a household’s assets^1^. These were grouped into quintiles and provide a relative measure of wealth. Place of residence (urban or rural) is also provided by the survey.

Children born in the five years prior to the survey were included in the analysis. The three surveys allow us to look at both short and medium term trends to enable a more nuanced analysis of progress. Countries were grouped separately for the two indicators used: presence of a skilled attendant at the birth (SBA), and whether sufficient antenatal care (ANC) visits were undertaken, defined as having four or more visits before the birth. These indicators were chosen as they are both key components of maternal health care and data is available from all DHS over the time period of the study; in addition both SBA and ANC are indicators used to monitor progress towards MDG 5. Ideally postnatal care would also have been included, but availability of data on this indicator is limited. Groups were defined by the overall national coverage level of the respective indicator in the most recent survey: very low (<30 %), low (30 %-49 %) moderate (50 %-64 %), high (65 %-79 %) and very high (80 % or above).

The results are based on simple percentages of women who had sufficient ANC and had a SBA within each income group and place of residence. All analyses were weighted to account for the differential chances of individuals being selected into the sample. Changes over time are measured by the average annual change in percentage points. Annual percentage rate of change was not used as this can show misleadingly sharp improvements for countries with a very low baseline.

## Results

### How great is the current gap between rich and poor for maternal health services?

**Fig. 1:**
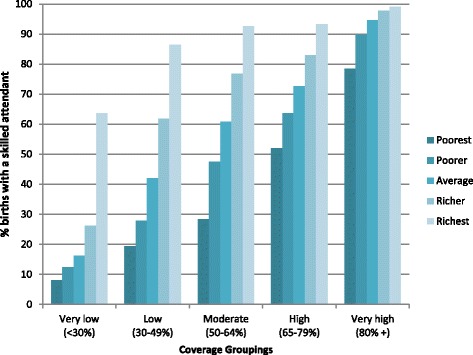
SBA by wealth quintile grouped by overall coverage rate for 35 countries using data from DHS surveys (most recent available survey 2006–2012). Country groupings are: Very low: Ethiopia, Mali, Bangladesh, Niger. Low: Haiti, Nigeria, Nepal, Madagascar, Kenya, Zambia, India, Tanzania, Guinea. Moderate: Senegal, Mozambique, Uganda, Cote D’Ivoire, Ghana, Philippines, Cameroon. High: Zimbabwe, Burkina Faso, Rwanda, Bolivia, Cambodia, Malawi, Egypt. Very high: Namibia, Indonesia, Benin, Peru, Columbia, Dominican Republic, Jordan, Armenia

**Fig. 2:**
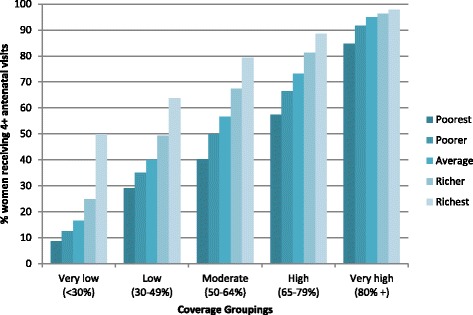
At least four ANC visits coverage by wealth quintile grouped by overall coverage rate for 35 countries using data from DHS surveys (most recent available survey 2006–2012). Country groupings are: Very low: Ethiopia, Bangladesh. Low: Niger, Burkina Faso, Rwanda, Mali, India, Tanzania, Cote D’Ivoire, Malawi, Senegal, Kenya Uganda, Nigeria, Madagascar. Moderate: Nepal, Mozambique, Guinea, Cambodia, Zambia, Benin, Cameroon. High: Zimbabwe, Egypt, Haiti, Bolivia, Namibia, Philippines, Ghana. Very high: Indonesia, Columbia, Peru, Jordan, Dominican Republic, Armenia

Out of the 35 countries included in the study only eight have reached the 80 % coverage target for national level SBA coverage, and six for ANC. Six countries have achieved 80 % coverage for both services (Indonesia, Jordan, Namibia, Colombia, Armenia and Peru). Figures 1 and 2 show the coverage level for SBA and ANC based on the most recent data available from the DHS, grouped based on the overall level of national coverage at the time of the most recent survey and also by wealth quintile. While SBA coverage is limited for all women in the groups with very low or low coverage, the poorest quintiles still have markedly less access, and the ratio between coverage for the highest and lowest quintile (Q5:Q1: a common indicator of inequality) is markedly greater than for those groups with higher coverage. A woman in the poorest 20 % from the very low SBA group is eight times less likely to deliver with a SBA than her counterpart in the wealthiest 20 % (Q5:Q1 = 8.0). While the differential diminishes as overall coverage increases, clear inequities continue in the groups with moderate and high coverage, and only really become minimal in the highest coverage group (Q5:Q1 = 1.3). If we look more closely at the group with “high” coverage of SBA (an overall rate of 65-79 %) these countries have achieved an overall average of 71 % coverage, so close to the 80 % target. However on average only 52 % of the poorest quintile received skilled care at birth. Even in those countries where the 80 % target has been achieved there are still inequalities between rich and poor – the richest group almost have 100 % SBA coverage, while the poorest quintile has not yet reached the 80 % threshold. The differentials for coverage between quintiles for four or more ANC visits are somewhat lower but there is still a difference of over 30 percentage points between richest and poorest in all groups of countries apart from very high coverage group.

**Fig. 3:**
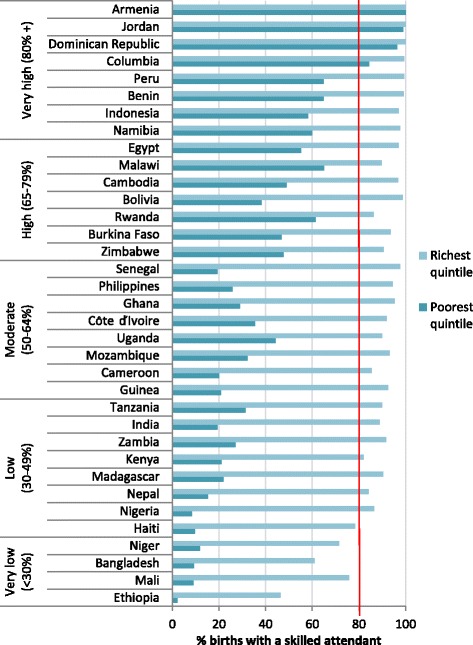
SBA coverage for poorest and richest quintiles in 35 countries (80 % coverage target marked in red: most recent available survey 2006–2012)

Figure 3 demonstrates the huge difference in the gap between current coverage and the 80 % target for SBA for the poorest and richest quintiles in the individual countries included in the analysis. Five of the six countries with the lowest coverage currently have less than 10 % of women in the poorest quintile accessing SBA: in Ethiopia the figure is only 2 %. Coverage for the poorest quintile remains below 50 % for countries in the low and medium coverage groups, and a number of countries in the high group. The poorest have reached the 80 % coverage target in only four countries (Dominican Republic, Jordan, Armenia and Colombia): these countries have achieved overall coverage of at least 90 %. Even in countries which have reached the 80 % target at national level the poorest may have very limited access to SBA: in Indonesia only 58 % of the poorest women receive skilled care at birth, and in Namibia the figure is 60 %. On the other hand, the richest quintile has already reached 80 % coverage in all but five countries, which are those with the lowest overall coverage.

### How great is the gap between urban and rural residents for maternal health services?

**Fig. 4:**
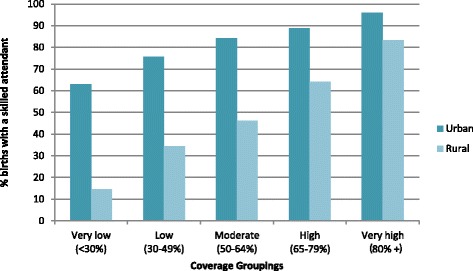
SBA coverage by place of residence grouped by overall coverage rate for 35 countries using data from DHS surveys (most recent available survey 2006–2012)

**Fig. 5:**
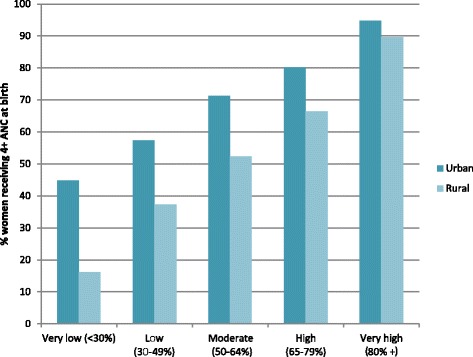
4+ ANC coverage by place of residence grouped by overall coverage rate for 35 countries using data from DHS surveys (most recent available survey 2006–2012)

Figures 4 and 5 show the differences in coverage for urban and rural dwellers for each coverage grouping. Again the differences are greatest for countries with the lowest coverage: the percentage of urban dwellers receiving skilled attendance in the “very low” category is more than four times that of their rural counterparts. Differences stay marked for the low, medium and high categories.

### What progress is needed to reach the 80 % coverage target by 2030?

As the 80 % target is absolute rather than relative, the progress required by countries with currently poor coverage is far greater than for those who have already achieved higher coverage. Table 1 shows the annual percentage increase in coverage required for both SBA and ANC in order to reach the 80 % coverage target disaggregated by wealth quintile using data from the most recent DHS as a baseline. The lowest coverage group countries will need to increase coverage each year by an average of 2.9 and 3.1 percentage points for SBA and ANC respectively at a national level. For the poorest quintile within the lowest coverage group the growth required is even greater: an annual increase of on average 3.6 and 3.8 percentage points is required for SBA and ANC. However, for the richest quintile all but the lowest coverage group have already reached the target for SBA, and all but the lowest two groups for ANC. Table 2 shows the progress required for the different coverage groups disaggregated by urban and rural residence. In the lowest coverage group service use amongst rural residents will need to increase by an average of 3.3 percentage points each year compared to a much more modest 0.9 % amongst urban dwellers for SBA; the differences are somewhat less for ANC. The progress needed by rural residents in the “high” coverage group for SBA (0.8 percentage points per annum) is only slightly less than that needed by the richest quintile of the poorest coverage group (0.9 % per annum).

**Table 1:** Required annual percentage point increase in coverage required to attain 80 % coverage by 2030, by coverage group and wealth quintile

SBA	Poorest	Poorer	Average	Richer	Richest	Overall
<30 %	*3.6*	*3.4*	*3.2*	*2.7*	**0.9**	*2.9*
30-49 %	*2.9*	*2.5*	*1.8*	**0.8**	-	*1.7*
50-64 %	*2.6*	*1.6*	**0.9**	**0.1**	-	*1.1*
65-79 %	**1.4**	**0.8**	**0.4**	-	-	**0.4**
80 % +	**0.1**	-	-	-	-	-
ANC						
<30 %	*3.8*	*3.6*	*3.3*	*2.9*	*1.6*	*3.1*
30-49 %	*2.5*	*2.2*	*2.0*	*1.5*	*0.8*	*1.8*
50-64 %	*2.1*	**1.6**	**1.2**	**0.6**	**0.0**	**1.2**
65-79 %	**1.1**	**0.7**	**0.3**	**0.0**	-	**0.4**
80 % +	-	-	-	-	-	-

Figures in bold indicate that growth is on track to meet the 80 % target by 2030; figures in italics indicate that growth is not on track to meet the 80 % target (based on longer term trends)

**Table 2:** Required annual percentage point increase in coverage required to attain 80 % coverage by 2030, by coverage group and place of residence

SBA	Urban	Rural	Overall
<30 %	*0.9*	*3.3*	*2.9*
30-49 %	**0.2**	*2.2*	*1.7*
50-64 %	-	*1.7*	*1.1*
65-79 %	-	**0.8**	**0.4**
80 % +	-	-	-
ANC			
<30 %	*1.9*	*3.4*	*3.1*
30-49 %	*1.1*	*2.1*	*1.8*
50-64 %	**0.4**	*1.6*	**1.2**
65-79 %	-	**0.6**	**0.4**
80 % +	-	-	-

Figures in bold indicate that growth is on track to meet the 80 % target by 2030; figures in italics indicate that growth is not on track to meet the 80 % target

To establish how required progress compares with past progress we examined past trends in coverage. Tables 3 and 4 show the average annual percentage increase in coverage for SBA and at least four ANC visits over two different time periods by quintiles. The first is growth over a period of more than a decade, calculated using the most recent survey and a survey at least 10 years previously. The second period is shortened, calculating the growth between the most recent two surveys, with a mean period of 5.6 years. Growth has been faster in the most recent period for most quintiles than over the longer timescale for SBA in the very low, low and medium coverage countries. In the high coverage group the percentage of women receiving SBA increased fastest for the most recent period for the poorest and poorer groups, while it has recently slowed down for the other wealth quintiles. For the richest quintile growth was fastest across all quintiles in the longer time period. For ANC there are mixed results, although in general growth in the percentage receiving sufficient care has recently slowed or is stagnant.

**Table 3:** Average annual percentage point change between two surveys for SBA and ANC: disaggregated by wealth quintiles

	Average time period 11.9 years						Average time period 5.6 years					
SBA	Poorest	Poorer	Average	Richer	Richest	Overall	Poorest	Poorer	Average	Richer	Richest	Overall
<30 %	0.3	0.3	0.4	0.5	1.0	0.4	0.2	0.4	0.5	1.1	1.8	0.6
30-49 %	0.2	0.6	0.9	1.1	0.9	0.6	0.5	0.8	1.7	1.7	1.1	1.1
50-64 %	0.7	1.2	1.1	0.7	0.6	0.8	0.6	2.1	1.4	0.5	0.7	1.1
65-79 %	2.3	2.7	2.8	2.6	1.0	2.3	3.1	3.6	3.1	2.6	1.0	2.8
80 % +	1.5	1.5	1.1	0.9	0.4	1.2	1.5	1.3	0.8	0.5	0.1	0.9
ANC												
<30 %	0.5	0.7	1.0	1.3	1.2	0.9	0.5	0.9	1.0	1.3	1.3	1.0
30-49 %	0.3	0.3	0.4	0.5	0.5	0.4	0.2	0.8	0.8	0.9	0.2	0.6
50-64 %	1.1	1.6	1.7	1.6	1.1	1.5	1.1	1.6	1.5	1.5	0.7	1.3
65-79 %	1.7	1.8	1.6	1.5	0.9	1.6	1.8	1.8	1.4	1.0	0.2	1.3
80 % +	2.3	1.8	1.0	0.7	0.5	1.4	2.3	1.9	1.1	1.0	0.4	1.3

**Table 4:** Average annual percentage point change between two surveys for SBA and ANC: disaggregated by place of residence

	Average time period 11.9 years			Average time period 5.6 years		
SBA	Urban	Rural	Overall	Urban	Rural	Overall
<30 %	0.7	0.3	0.4	0.8	0.3	0.6
30-49 %	0.8	0.6	0.6	1.4	0.9	1.1
50-64 %	0.2	0.8	0.8	0.4	1.4	1.1
65-79 %	1.2	2.2	2.0	-	3.2	2.8
80 % +	0.6	1.5	1.2	-	1.2	0.9
ANC						
<30 %	0.6	0.9	0.9	−0.1	0.8	1.0
30-49 %	0.3	0.3	0.4	0.4	0.5	0.6
50-64 %	1.0	0.8	1.5	1.1	0.9	1.3
65-79 %	1.1	1.9	1.6	0.7	1.7	1.3
80 % +	0.7	2.1	1.4	0.8	2.2	1.3

Unsurprisingly given their current status, progress has been much smaller in the very low, low and medium coverage groups for SBA and the very low and low coverage groups for ANC than is required to reach the 80 % target. For the lowest coverage groups there is a marked wealth gradient, with much less progress being made in the poorer than the richer quintiles. Even comparing against more recent trends in coverage increase indicates that the countries in the very low and low coverage groups are still off-track and will not achieve 80 % coverage unless there is a large increase in the rate of growth for coverage.

In terms of urban and rural residence, progress in rural areas has been slower in the two lowest coverage groups, for SBA, but interestingly has been greater or the same for ANC in these two groups. This might reflect the fact that ANC is often easier to roll out, as doesn’t require 24 h cover, and can be provided at a lower level of the health system. Progress in rural areas is not on track to reach 80 % by 2030 for very low, low or moderate coverage groups.

**Fig. 6:**
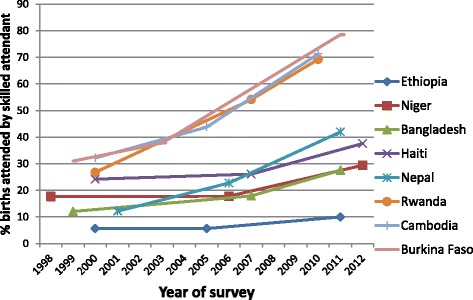
Progress in SBA for countries with <30 % coverage at baseline survey based on DHS data

Grouping countries by their current level of achievement is useful in that it enables us to understand how coverage needs to be accelerated compared to past progress, but it does not inform us whether there are examples of rapid progress in countries starting from a low baseline. Therefore in Fig. 6 we show the eight countries that have the lowest coverage at the baseline survey (ranging from Ethiopia with 6 % coverage to Cambodia with 32 % coverage). Ethiopia has made little progress and is still in the very low coverage group. Four others have made some progress more recently, although Niger and Bangladesh still have SBA coverage of under 30 % overall. Three further countries, Rwanda, Burkina Faso and Cambodia, have made striking progress. In Rwanda coverage has increased by 4.2 percentage points per annum: the poorest quintile coverage has increased by 4.6 percentage points per annum over 10 years, and 5.7 percentage points over the most recent three years. Cambodia has increased coverage over the last 10 years at a rate of 3.9 percentage points per annum, but this has risen to a rate of 5.5 percentage points (and 5.7 percentage points for the poorest quintile) per annum between 2005 and 2010. This suggests that while unusual, the progress needed to reach the 80 % target is not unprecedented even amongst the poorest groups from countries with extremely low initial coverage.

### Combining the urban/rural and socio-economic dimensions

The WHO 80 % target states that this should be achieved irrespective of wealth or location. The previous analysis treats these dimensions as separate, making it difficult to compare attainment by socioeconomic status within urban and rural areas. Simply disaggregating urban and rural areas by wealth quintile offers a limited contribution to the measurement of inequalities. The wealth measure used within the DHS consists overwhelmingly of rural residents in most countries, simply as an artifact of its construction as more rural than urban dwellers are usually interviewed. This can be seen in the example of Kenya which we present in Table 5. It shows the lack of association between people identified as the poorest within urban areas and those identified nationally. For those who are in the poorest 20 % of urban dwellers only about 2 % are classified as poorest over the whole country. Almost 100 % of those who are in the average or higher quintiles, when calculated using urban dwellers only, are in the wealthiest quintile when calculated over the whole country. Hence potential lack of progress in the poorest urban group may be hidden both by quintiles (where lack of progress may be masked by improvements for rural residents) and urban/rural residence (where lack of progress may be masked by increased access for more wealthy urban residents). Disaggregating wealth within both rural and urban areas will provide a more accurate picture of groups that are progressing well or falling further behind in moving towards UHC.

**Table 5:** Percentage of women in each national wealth quintile within quintiles calculated from urban residents only: Kenya

	National Wealth Quintiles				
Urban Only Wealth	Poorest	Below Average	Average	Above Average	Wealthiest
Poorest	2.3	5.8	8.7	51.9	31.2
Below Average	0.0	0.0	3.4	26.6	70.0
Average	0.0	0.0	0.0	3.5	96.5
Above Average	0.0	0.0	0.0	0.0	100.0
Wealthiest	0.0	0.0	0.0	0.0	100.0
Overall	0.6	1.5	3.0	19.7	75.3

**Fig. 7:**
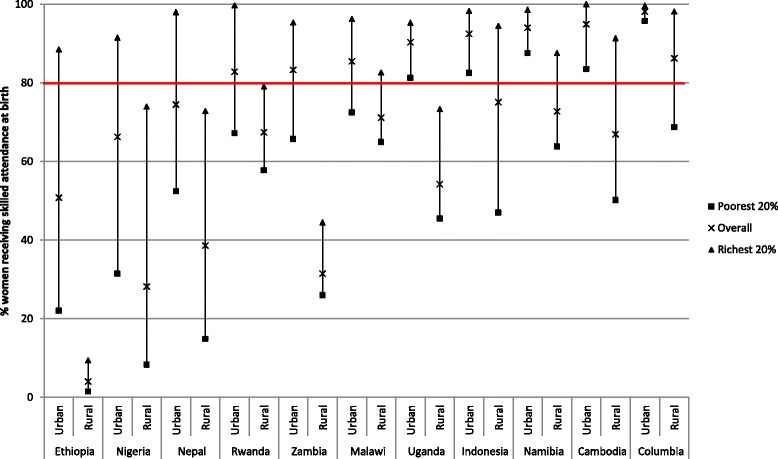
SBA coverage for urban and rural residents, split by wealthiest and poorest quintiles for selected countries based on DHS data (most recent available survey 2006–2012)

Figure 7 shows the percentage of women with a skilled birth attendant at the latest time point, split by urban/rural and for the richest and poorest quintiles within place of residence. Ten countries have been selected in order to show the disparities within and between place of residence. Wealth has been calculated, using the standard PCA analysis, for urban and rural areas separately; by doing this an accurate view of inequalities by place of residence are revealed. Firstly it is clear that there are large differences in coverage between urban and rural areas in all countries. In urban areas 7 out of the 10 countries have coverage above 80 %; in rural areas only 1 country passes this threshold. However the poorest in urban areas are often lagging badly, with coverage far lower than their richest counterparts. In some countries the richest rural dwellers have higher access to a SBA than poor urban residents, while in others all rural women, irrespective of wealth, have extremely poor access in comparison to urban women. This is seen when countries have medium to high levels of coverage across the whole country which has increased over a relatively long period of time.

## Discussion

The rate of progress needed for the countries with lowest coverage to reach the 80 % at the national aggregate level is extremely challenging, but not completely unprecedented at an individual country level. We have already provided the examples of Cambodia and Rwanda, both of whom have exceeded the required percentage increase needed by countries with the poorest coverage to reach 80 % by 2030 for both the country as a whole and those in the poorest quintile. While no other countries have reached the required level of increase over the medium term, several countries have demonstrated rapid growth over recent years. Burkina Faso and Uganda have both achieved rates of progress of over 3 percentage points a year for both the whole country and the poorest quintile for the period between the last two surveys for SBA. With the exception of Cambodia no countries have achieved the required rate of progress for ANC across all three surveys, but several countries such as Nepal and Egypt have made progress at a national level which is almost sufficient (3.6 % and 3.0 % respectively), although progress is much slower for the poorest quintile. Rigorous analysis should be carried out to ascertain the drivers behind progress in these countries, particularly when the gains embrace the poorest, and lessons learnt should be collected and widely disseminated.

The UHC goal is an absolute rather than a relative target: countries with the lowest coverage will need to make the most progress. These are countries where the infrastructure is weakest, and attempts to increase coverage of key MNH interventions will require health system strengthening and in particular a massive focus on developing a workforce that can provide an adequate level of care to women and their babies. This will require substantial investment, and donors and national governments will need to ensure funding is adequately allocated and focussed.

In many countries, the progress needed to reach 80 % is quite modest for the more wealthy or urban populations, but needs to be much greater for the poorest and those in rural areas. A key question is whether progress to UHC for maternal health care services can be achieved without increasing existing inequalities. Channon et al. [6] highlight that all countries in the past have experienced similar patterns in inequity on the road from low to high coverage of maternal health interventions: inequities increase initially when starting from a very low baseline, but decrease as overall coverage increases. However, what is clear is that some countries have transitioned more quickly through the patterns of inequitable access than others, and lessons can be learnt on how maternal health services can be developed in a way to minimise disparity.

### Ensuring access for the poorest mothers

The concept of universalism in health care coverage is often used to indicate impartiality in the provision of services and allocation of resources, and suggests that “equal” treatment of different population groups will result in increased equity. However, such approaches may exclude specific groups by failing to recognise their specific needs, and insufficient efforts to deal with diversity may produce a “false universalism” that does little to reduce inequalities [14]. Efforts to target the disadvantaged are commonly used to overcome this limitation, but effectively identifying, specifying and reaching the appropriate groups to target is a complex issue.

A number of writers [15–18] suggest that efforts to target interventions to the poorest can help in reducing inequities while moving towards UHC, and indeed evidence suggests that countries making rapid progress in overall coverage show improvements in equity for skilled attendance [19]. However the literature on how services can best promote pro-poor coverage is sparse for maternal health care. The bulk of existing studies have focussed on reducing financial barriers through either targeted or universal interventions such as insurance, conditional cash transfers, voucher schemes and removal of user fees [20–22].

What is less well understood is how maternal health care can be organised and delivered to best promote equitable access, although measures to improve coverage will need to address both supply and demand factors beyond financing. Outreach approaches that provide services for women through the development of community based MCH cadres are often seen as a key component of pro-poor maternal health care services, but with a few exceptions [23, 24] they are rarely evaluated from an equity perspective. In addition, such interventions often lack the necessary structures and processes to ensure adequate access to emergency obstetric care [25], so evaluation of one component of the continuum may fail to identify whether such services are sufficient to make a real difference for women and their infants. Few studies have examined the impact of programmes on increasing demand for services from an equity perspective [24], although a number of such programmes focussed within poor communities have been evaluated with mixed results [26]. Further analysis is needed on pro-poor approaches to developing maternal health services, and a comprehensive review of existing evidence as well as where relevant new research should be used to guide and develop policy. Opportunities lie in greater analysis of the approaches used by countries that have achieved rapid and equitable coverage for maternal health and the factors that have facilitated this progress.

### How should progress with equity be monitored?

Over the years a number of approaches to capturing the dynamics of inequitable access have been developed and proposed. While more complex measures may offer insight into the dimensions of inequality, they often have the disadvantage that they are more difficult to understand and utilise for a generalist audience and are difficult to compare across time and place. Our study clearly highlights the importance of measuring equity concurrently by place of residence and wealth. Access is normally measured by urban/rural residence and wealth separately but this fails to capture the interactions between these two factors. We strongly recommend that wealth quintiles are measured separately for urban and rural residents. The poor in both rural and urban areas are the most poorly served for health services, and the level of their disadvantage, especially amongst the urban poor, is often underestimated in overall national quintiles.

### Limitations of the study

This study has a number of limitations which are worth noting. The analysis is dependent on the accurate reporting of indicators, which, particularly in the case of skilled attendance at birth, may be somewhat problematic. This indicator relies on a woman being able to identify and report the cadres of health care worker who attended the birth, but this has been shown to be unreliable [27]. In addition, while there is a strict definition of the competencies needed for a health care worker to be assigned as a “skilled attendant” [28] this cannot be easily verified: specific cadres are assigned as skilled attendants within each country, but this may not actually reflect their skills. Harvey et al. [29] found a high proportion of health care workers from cadres considered “skilled” did not have the necessary knowledge and competencies required by the WHO definition. A further problem is that we were not able to discuss differences between urban and rural residence in more depth: the category of rural (which is not universally defined) can range from peri-urban to extremely remote, and our somewhat simplistic analysis based on available classification may mask more complex patterns. A final limitation is the trends over time by quintile assume that the urban/rural composition of the countries is static over the time period analysed. When calculated over the whole country the wealth indices are affected by these compositional factors. The potential scale of these effects is impossible to estimate.

## Conclusion

For many countries which currently have poor levels of access to maternal health services reaching the target of 80 % coverage, even at national level, will be challenging. For other countries this target is not challenging enough and the target of true universal coverage – 100 % for all groups – should be set in order to drive access forward further. It has been shown that in many countries with good overall national coverage the poor and the rural residents are still left behind. Ensuring universal coverage is achieved for the poorest and those living in rural areas is a challenge that will require sustained commitment from Governments, donors and international policy makers, although there is little evidence about the best ways to do this. Lessons must be carefully gathered, learnt and disseminated from countries that have made fast and equitable progress in order to effectively focus efforts and resources. The 80 % target overall is a positive step to drive the UHC agenda, but needs to be carefully managed and measured in order that progress with equity across all groups is achieved.

## Appendix

**Table 6:** Countries included in the study with survey years used

Country	Survey 1	Survey 2	Survey 3 (Most Recent)
Armenia	2000	2005	2010
Bangladesh	1999	2007	2011
Benin	1996	2006	2011
Bolivia	1994	2003	2008
Burkina Faso	1998	2003	2011
Cambodia	2000	2005	2010
Cameroon	1998	2004	2011
Columbia	2000	2005	2010
Cote d’Ivoire	1998	2005	2011
Dominican Republic	1996	2002	2007
Egypt	1995	2005	2008
Ethiopia	2000	2005	2011
Ghana	1998	2003	2008
Guinea	1999	2005	2012
Haiti	2000	2005	2012
India	1992	1998	2005
Indonesia	1997	2007	2012
Jordan	1997	2007	2012
Kenya	1998	2003	2008
Madagascar	1997	2003	2008
Malawi	2000	2004	2010
Mali	1995	2001	2006
Mozambique	1997	2003	2011
Namibia	1992	2000	2006
Nepal	2001	2006	2011
Niger	1998	2006	2012
Nigeria	1999	2004	2008
Peru	2000	2004	2012
Philippines	1998	2003	2008
Rwanda	2000	2007	2010
Senegal	1997	2005	2012
Tanzania	1999	2004	2010
Uganda	2000	2006	2011
Zambia	1996	2001	2007
Zimbabwe	1999	2005	2010

## Abbreviations

ANC: Antenatal care
SBA: Skilled birth attendant
DHS: Demographic and household survey
MDGs: Millennium development goals
SDGs: Sustainable development goals
UHC: Universal health care

## Footnotes

1 Where provided within each country dataset the wealth index was used. In countries where this was not provided these were calculated using the methodology specified by Rutstein and Johnson in ‘The DHS Wealth Index’ (DHS Comparative Reports No.6).
